# Modelling biological behaviours with the unified modelling language: an immunological case study and critique

**DOI:** 10.1098/rsif.2014.0704

**Published:** 2014-10-06

**Authors:** Mark Read, Paul S. Andrews, Jon Timmis, Vipin Kumar

**Affiliations:** 1 Department of Electronics, University of York, York YO10 5GW, UK; 2 Department of Computer Science, University of York, York YO10 5GW, UK; 3 Torrey Pines Institute for Molecular Studies, San Diego, CA 92121, USA

**Keywords:** computational biology, diagrammatic modelling, modelling and simulation, unified modelling language, immunology

## Abstract

We present a framework to assist the diagrammatic modelling of complex biological systems using the unified modelling language (UML). The framework comprises three levels of modelling, ranging in scope from the dynamics of individual model entities to system-level emergent properties. By way of an immunological case study of the mouse disease experimental autoimmune encephalomyelitis, we show how the framework can be used to produce models that capture and communicate the biological system, detailing how biological entities, interactions and behaviours lead to higher-level emergent properties observed in the real world. We demonstrate how the UML can be successfully applied within our framework, and provide a critique of UML's ability to capture concepts fundamental to immunology and biology more generally. We show how specialized, well-explained diagrams with less formal semantics can be used where no suitable UML formalism exists. We highlight UML's lack of expressive ability concerning cyclic feedbacks in cellular networks, and the compounding concurrency arising from huge numbers of stochastic, interacting agents. To compensate for this, we propose several additional relationships for expressing these concepts in UML's activity diagram. We also demonstrate the ambiguous nature of class diagrams when applied to complex biology, and question their utility in modelling such dynamic systems. Models created through our framework are non-executable, and expressly free of simulation implementation concerns. They are a valuable complement and precursor to simulation specifications and implementations, focusing purely on thoroughly exploring the biology, recording hypotheses and assumptions, and serve as a communication medium detailing exactly how a simulation relates to the real biology.

## 1. Introduction

Computational modelling and simulation methods are increasingly employed as a complement to traditional wet-laboratory techniques in exploring biological processes such as those of the immune system [1]. Computer simulators that combine experimental data with theories of system operation and composition offer a flexible means by which to test hypotheses, perform preliminary exploratory experiments and potentially guide subsequent laboratory experimentation [2–4].

The construction of a well-engineered simulator that models the behaviour of a complex biological system is a non-trivial exercise, which will often involve an interdisciplinary collaboration between a biologist (the domain expert) and a computer systems engineer responsible for the implementation of the simulator code [5]. Communication between these parties is key in order that a sensible model of the biology is implemented in a sensible manner. Likewise, communication of the biological model underlying the simulator to the wider community helps to ensure a healthy research field. This process of *domain modelling* helps establish what is part of the model, and just as importantly what is not part of the model, and is essential for correct interpretation of simulation results, and assessing their contribution in the wider biological research context.

The role of a *domain model* is to present a coherent and transparent model of a biological system. It captures assumptions, abstractions and hypotheses made of a biological system, which can arise in a variety of manners. It is often unclear which biological factors are implicated in a particular biological phenomenon of interest, and furthermore, it is both computationally and conceptually intractable to capture in simulation every aspect of a biological system, and assumptions (often implicit) are made to address this. The process of constructing a domain model entails a thorough exploration of the biology, examining the biological system from a variety of perspectives, including low-level components such as cells or, conversely, top-down perspectives of how system-level behaviours emerge. Domain modelling can highlight inconsistencies in available data, or a lack thereof, which again necessitate that assumptions and hypotheses be adopted. A domain model captures how system-level behaviours are hypothesized to manifest from the mass action of low-level components such as cells, and these hypotheses may be subjected to evaluation in subsequent simulation.

**Figure 1.**
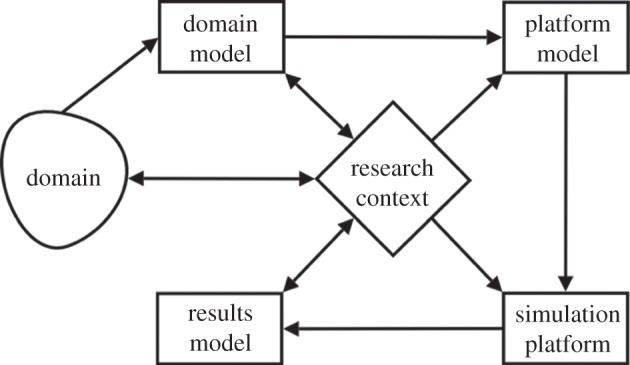
An overview of the artefacts comprising the CoSMoS process, and the flow of information between them. Adapted from [7].

Domain modelling is a key element of our wider strategy for engineering high-quality simulations for use in exploring biological domains: the ‘CoSMoS process’ [6–8], further reviewed in the electronic supplementary material and summarized in figure 1. CoSMoS comprises several modelling activities and artefacts intended to capture, justify and explicitly document the assumptions made of a biological system in the various stages of simulator development and use. The biology under study is referred to as the *domain*. The domain modelling stage intentionally omits any simulation implementation concerns, and the resultant domain model is non-executable; it focuses purely on what is known of the biology, not how it is to be implemented. Implementation-specific information, assumptions and constructs are recorded in the *platform model*, a subsequent CoSMoS modelling phase that builds on the domain model and yields a software specification from which a simulation can be constructed. A domain model can be simulated in a number of different ways, and each possible strategy for implementation is accompanied by its own set of assumptions. Furthermore, the hypotheses and emergent properties explicitly captured in the domain model should not appear explicitly in any software specification; these emergent properties should be observed as high-level simulation behaviours in the same manner that they are in the domain, and hypotheses are to be tested based on simulation results. Neither should be directly coded into the simulation. These reasons necessitate the separation of domain and platform models; the former dealing with scientific concerns in the domain, the latter concern with issues relating to implementation. An example of the separation between domain and platform models may be found in the supporting materials. The platform model is implemented as a *simulation platform* which can be executed and facilitates computer-based experimentation, from which results are recorded (as the *results model*) and interpreted in the context of the biology. This may entail evaluating hypotheses or making predictions.

The CoSMoS process is intended to be an iterative process, where each of the models undergoes potential modification between iterations. An iteration is envisaged to comprise three stages: *discovery* where the domain model is modified to reflect further investigation of the domain or changes to the context in which research is to be conducted; *development* in which the platform model and simulation platforms are updated to reflect the modified domain model; and *exploration*, where simulation experiments are performed. If the simulation fails to adequately capture the biology, then further iterations of the CoSMoS process are warranted, which may begin with re-examining the biology and amending the domain model, and then tracing these amendments through the other CoSMoS artefacts.

In this paper, we describe a framework for creating a domain model, which captures and communicates biological concepts as a collection of diagrams at various levels of scope and abstraction. The tool chosen for creating the diagrams advocated in our domain modelling framework is the unified modelling language (UML) [9]. The UML is a collection of diagrammatic notations allowing for a wide range of specification scopes, from high-level system overviews to low-level focuses on particular system components [9,10]. It can represent both static and dynamic views of a system, at various levels of abstraction. Static views depict the relationships that components hold with one another, whereas dynamic views express the collaborations between system components and the changes to their internal states that influence their behaviours. This ability to specify a system from different views has made the UML a popular modelling tool, and it finds application outside of the software domain within which it was conceived [10].

Here, we discuss advantages and disadvantages of using elements of the UML to apply our framework in the context of a domain model of experimental autoimmune encephalomyelitis (EAE) [11–13], a mouse model of multiple sclerosis [14]. Immunological phenomenon has been subjected to exploration by simulations ranging from receptor modelling [15], organ-level modelling [16,17], to systemic disease [4,18,19]. The EAE case study highlights the spatial and temporal scales of complexity seen throughout biology whereby systemic behaviours manifest from the interactions of millions of cells and signalling molecules, of differing types, in various heterogeneous spatial compartments. Networks of cells and molecules comprise numerous positive and negative feedback pathways, which can result in either stable system-level behaviours, or frequent switches. Both the EAE domain model and its subsequent simulation are published in full, as open access, elsewhere [4]. Here, the domain model is used to illustrate our domain modelling framework, and present a critique of modelling EAE with the UML.

The appeal of diagrammatic modelling to biologists stems from its approachability compared with other methods (e.g. equation-based) [2]. The perceived parallels with the manner in which biologists reason about the systems have led to the promotion of UML as a tool for the expression of agent-based simulation behaviours [20,21], and Harel statecharts (upon which UML state machine diagrams are based) were originally put forward for the modelling of complex systems [22]. Alternatives to the UML for graphically expressing biological systems include the systems biology graphical notation [23], which attempts to rectify the ambiguities and lack of universal applicability of existing notations for representing, primarily, biochemical systems. There is no universal standard accepted by the wider computational biology community [23], and we consider no modelling formalism to be superior to all others across all biological domains. Modellers should select the most appropriate modelling tool for the given problem. The UML is chosen here for its ease of abstraction that coincides with the multiple layers of abstraction in our domain modelling framework, and its successful record in representing multicellular multi-spatial biological systems [20,24–28].

Integrated technologies, such as IBM's rational rhapsody, the play engine and live sequence charts [29], can facilitate the diagrammatic specification and implementation of computer programs. They have been used to simulate biological systems such as the adaptive immune response generated within the lymph node with Harel statecharts [30] and *Caenorhabditis elegans* precursor cell fate with sequence diagrams [31]. These technologies have considerable appeal as integrated simulation specifications and implementations. They are, however, unsuitable for domain modelling as their executable nature necessitates the incorporation of implementation-specific assumptions and constructs that obscure the purely biological focus of a domain model.

Our paper is structured as follows. Section 2 describes our requirements for the modelling exercise, and is followed by an overview of EAE in which we discuss the usefulness of less formal diagrams that exist outside of the UML. Section 4 critiques the use of the UML's activity, class, sequence and state machine diagrams that have been applied to the description of various aspects of EAE. In §5, we show how the syntax of activity diagrams can be modified to better capture the nature of an immunological process such as EAE. Finally, we discuss the utility of the UML for modelling complex biological systems.

## 2. Domain modelling requirements

The model of EAE is to consist of a series of UML diagrams that capture and describe the biological processes of interest as they are currently understood by the EAE community, in particular our *domain expert* (co-author V.K.). The domain model should capture the scope, structure and behaviours of the domain using various diagrams as appropriate to represent different elements of the biology. The diagrams as a whole represent the system behaviours we are wanting to explore and simulate.

The domain model should coherently and transparently capture any assumptions and abstractions made of the biology. These arise from, and are necessitated by, the lack of understanding in biological systems (it is often this lack of understanding that motivates the simulation effort in the first place). The domain model helps to make the initial interpretation of the biology explicit before further abstractions are made during implementations.

In order to fulfil our EAE domain model requirements, we have developed a modelling framework that helps us describe it both systemically and from the view of individual actors. The framework defines three levels of modelling, with each level populated by multiple diagrams capturing a behavioural subset from a particular viewpoint:

*System*: the top modelling layer captures a system-level overview of the domain model components, how their interactions integrate to provide high-level behaviours and how these high-level behaviours are believed to contribute to phenomena observable in the real-world domain. This layer specifies which aspects of the biology are to be incorporated into the model, because only a subset of the entire real-world domain can be represented.
*Perspectives*: the second modelling layer represents a decomposition of system-level phenomena into various *perspectives*. System-level patterns, such as disease onset or resolution, are identified and their manifestation from component-level interactions depicted.
*Single-entity*: the third and lowest layer of modelling concerns the specification of single-entity-level dynamics in which the physical low-level entities of the system, such as cells and molecules, are considered as individuals and their behavioural dynamics specified.

Assumptions made of the biology can be captured in a number of ways. The diagrams created in accordance with our framework capture what of the biology is represented in the model, and textual accompaniment can highlight how known key aspects of the biology have been abstracted. Note, however, that it is impossible to create an exhaustive list of what is not represented in a model, as the field of biology is too large and new discoveries are made constantly. For this reason, we focus on capturing which elements of a biological field *are* represented, rather than which *are not*.

In the sections that follow, we explore the use of the UML and alternatives for capturing EAE with reference to expressing the three levels of modelling comprising our framework.

## 3. The domain: experimental autoimmune encephalomyelitis

**Figure 2.**
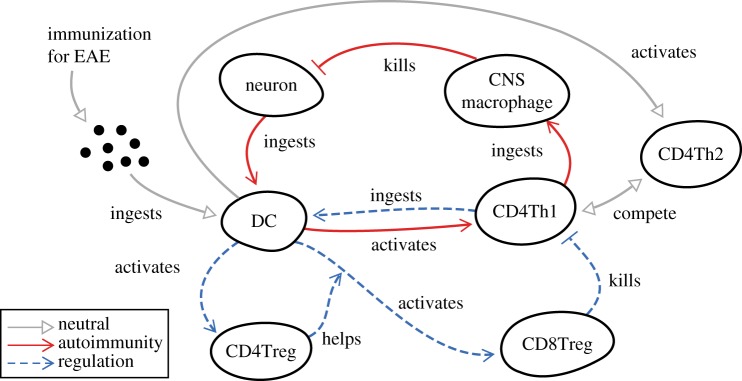
Abstract depiction of the major cell types involved in EAE autoimmunity and its associated recovery. Solid red arrows indicate interactions leading to autoimmunity, dashed blue arrows indicate regulatory activity that counters the autoimmune response, grey arrows are neutral. CD4Treg, CD8Treg, CD4Th1 and CD4Th2 cells are all varieties of T cell. Adapted from [4]. (Online version in colour.)

EAE is an autoimmune disease in mice that acts as a model of multiple sclerosis [14] in humans. EAE results in neurons of the central nervous system (CNS) being killed, causing paralysis. The cells involved in this particular EAE disease model are summarized in figure 2. Animals induced into EAE autoimmunity experience a period of partial paralysis, after which they spontaneously recover [12,13].

At the centre of this disease lie the immune cells called dendritic cells (DCs), T cells and macrophages. DCs ingest intercellular debris and other cells throughout the body and process them into materials recognized by certain varieties of T cells, resulting in T cell activation. The autoimmune paralysis-inducing stage is mediated through a cascade of cellular interactions that result in CNS-resident macrophage cells killing neurons. The recovery stage of EAE involves CD4Treg and CD8Treg cells (both varieties of T cell), the latter of which kill autoimmunity-inducing CD4Th1 cells (another variety of T cell) and interrupt the autoimmunity cascade. This paper contains only select examples of the full EAE domain model, used to demonstrate concepts. The full domain model, and a comprehensive review of EAE, may be found in the supporting information of Read *et al.* [4] which describes the full implementation of EAE as a simulation. A non-exhaustive list of key assumptions made of the biology may be found in the electronic supplementary material.

Having provided a brief overview of EAE, it is appropriate to present the top level of modelling first as it captures the overall scope of the biology of interest. While figure 2 is a useful complement to the text description for illustrating to the reader an informal overview of EAE, this type of cartoon does not address our requirements or fit within our three levels of modelling outlined above. Looking into the UML, we have not found any of its diagram types to be suitable for this requirement. In the light of this, we fall back on less formal diagrammatic notions to capture the top-level overview.

**Figure 3.**
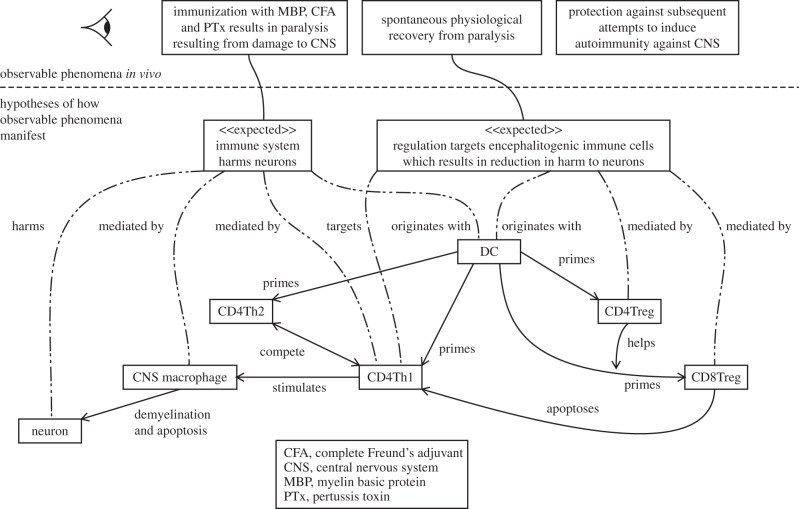
A research context diagram depicting the phenomena observed in the real domain, and the behaviours manifesting from cellular interactions believed to be responsible for them. Adapted from [4].

We have developed a diagram called the *research context diagram* to capture the top level of modelling for a complex biological system such as EAE (see figure 3 for the research context diagram for EAE). This diagram provides an immediate overview of the biology being modelled, highlighting the components hypothesized to play a significant role in the real-world phenomena and the system-level behaviours that result from their interactions. It does not have any formal semantics or syntax rules, and requires textual accompaniment to explain its use in a specific biological domain. The top of the figure details phenomena of interest in the biological system; the modelling and simulation endeavour is motivated by a desire to better understand them. Boxes annotated with ‘
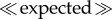
’ tags in the middle represent emergent system-level phenomena expected to manifest from low-level component (cellular, in this case) interactions. The components themselves, and an abstract indication of their interactions with one another, are represented at the bottom of the diagram. It is not claimed that other biological aspects not represented in the research context diagram are irrelevant, only that the abstractions represented on this diagram are sufficient for the emergence of phenomena observed in the real world.

**Figure 4.**
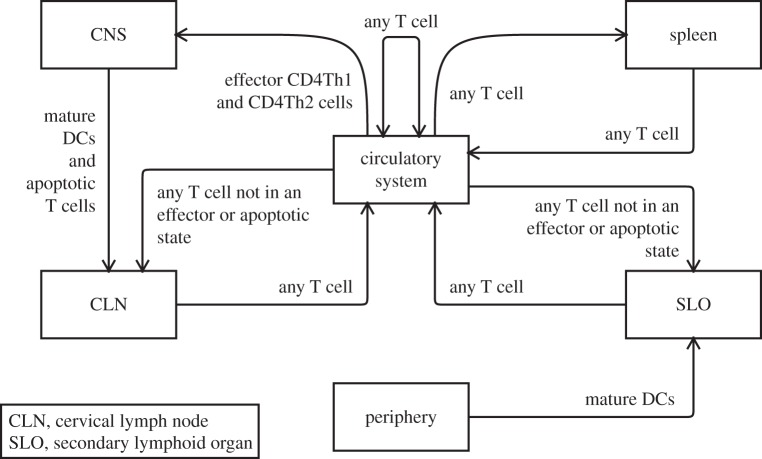
The spatial components of the domain model, and the manner in which the cells of the domain model may migrate between them. Adapted from [4].

For EAE, figure 3 shows how the immune system is hypothesized to be responsible for paralysis observed in mice induced into EAE, with DCs activating populations of CD4Th1 and CD4Th2 cells, the former of which stimulate CNS macrophages into harming neurons. The spontaneous recovery observed in mice is also hypothesized to be due to the immune system. DCs prime CD4Treg and CD8Treg populations, the latter of which kill CD4Th1 cells. The lack of a link from ‘protection against subsequent attempts to induce autoimmunity against the CNS’ serves to recognize the existence of this phenomenon, but asserts it to be beyond the current scope. The research context diagram does not describe the spatial aspects of EAE, which are separately represented in figure 4. This diagram complements the research context diagram, specifying the migration patterns of cells across the spatial compartments represented in the domain model.

## 4. Using the unified modelling language

The diagrams of the UML are useful in capturing the perspectives and single-entity levels outlined in our modelling requirements. Specifically, activity diagrams are well suited to capturing perspectives and state diagrams the single-entity dynamics. We explore here the use of these diagrams along with a discussion on the use of class diagrams and sequence diagrams.

### 4.1. Activity diagrams

Activity diagrams have provided a natural way to represent the mid-level perspectives of our modelling framework, as they allow any abstract concept to be represented as an activity, and can depict the order in which cellular events and interactions occur constituting a system-level behaviour. The perspectives represented by activity diagram expand on the research context diagram by decomposing complex system-level biological phenomena into stages that reveal greater detail, yet can still be captured on single diagrams. For example, EAE paralysis and subsequent recovery are represented as four phases: the initial inception of autoimmunity in the CNS following EAE induction; the snow-balling self-perpetuation of autoimmunity; the establishment of recovery-associated regulation, whereby CD4Th1 cells are killed by CD8Tregs; and the switch from an autoimmunity-inducing CD4Th1 immune reaction to a less harmful CD4Th2 variety. The former two expand upon the ‘immune system harms neurons’ expected behaviour in figure 3, and the latter two expand ‘regulation targets encephalitogenic immune cells which results in reduction in harm to neurons’.

#### 4.1.1. Capturing cell interactions

**Figure 5.**
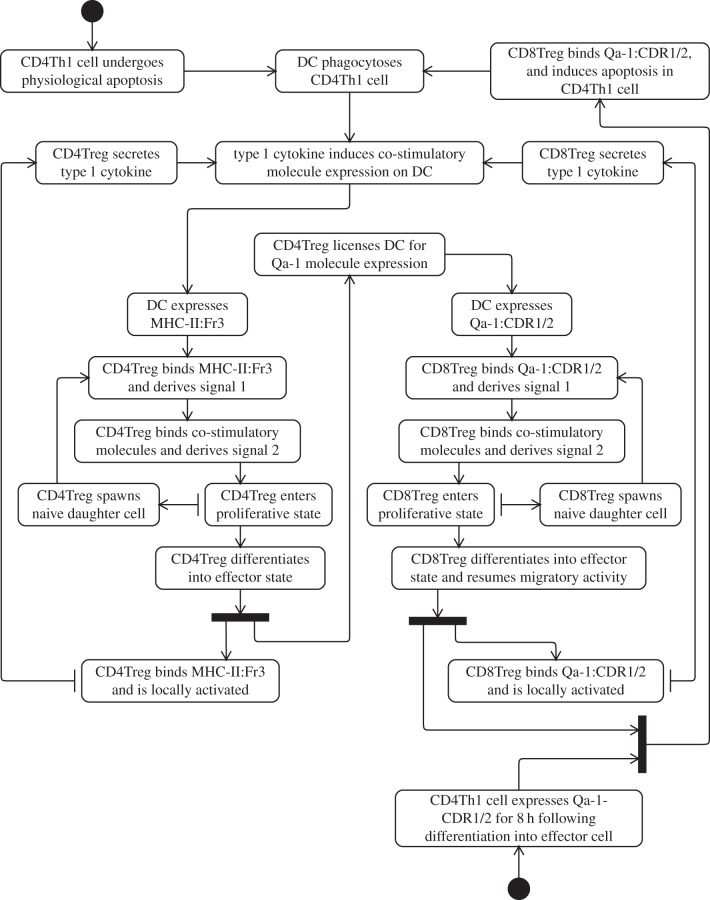
UML activity diagram depicting the cellular interactions and events that lead to the instigation and perpetuation of the regulatory immune response. Adapted from [4].

An example activity diagram showing the cellular interactions and events that lead to the instigation and perpetuation of the regulatory immune response is presented in figure 5. It demonstrates how autoimmunity-inducing CD4Th1 cells are ingested by DCs which activate CD4Treg and CD8Treg cells, the latter of which counter autoimmune activity. It has no end state, indicating the cyclic population-level events taking place; there is no clear singular point in time or cellular event that constitutes regulation having been established, it is a continuous and ongoing activity. The diagram also makes use of an additional syntax called the *propagating relationship* to depict the proliferative creation of daughter cells. An explanation of this relationship is presented later in §5.

Figure 5 is typical of our use of activity diagrams to capture the biological perspectives of our domain model. The activities in these diagrams are largely cellular interactions and state changes that take place in particular compartments of the domain model (such as those outlined in figure 4). Where appropriate, we use the UML ‘swim lane’ syntax (dotted lines) in the activity diagrams to segregate groups of activities according to the compartments in which they take place, an example of which is shown later in figure 14. The diagrams denoting our biological perspectives also differ from many other applications of activity diagrams by being cyclic in nature, and often do not contain end states. They have, however, been given start states to facilitate exploration of the diagram.

The major source of ambiguity in the use of activity diagrams to capture the biological perspectives arises from the multiplicity of cellular and molecular entities that interact and contribute to system-level behaviours in the real domain. The cellular events and interactions depicted as activities are mostly expressed at the single-cell level for clarity; however, for the perspective being modelled to occur, large numbers of cells must engage as captured. Additionally, perspectives should not be interpreted as implying that all cells exhibit the behaviours indicated; cells are highly stochastic and different individuals of the same type may exhibit very different dynamics. For the behaviours shown in the diagrams to be expressed by the biological system may require that sufficiently many, but not necessarily all, cells perform the activities indicated on the diagrams. Additionally, the cells engaging in the activities depicted do not necessarily do so simultaneously; at each point in time, there may be many populations of cells undertaking each of the activities depicted on the diagrams.

#### 4.1.2. Inability to represent compounding concurrency

**Figure 6.**
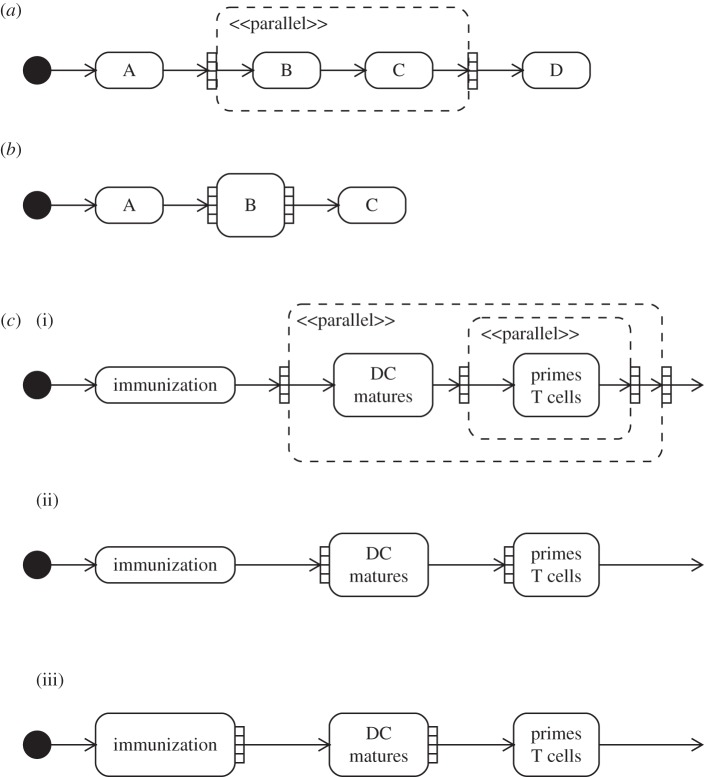
Examples of activity diagram expansion regions, and their potential application in depicting compounding concurrency. (*a*) An activity diagram expansion region. (*b*) An activity diagram compact expansion region. (*c*) Activity diagram expansion regions applied to a hypothetical perspective from the EAE domain.

To attempt to address the ambiguity of biological entity multiplicity, we have examined the use of UML activity diagram expansion regions for depicting ‘compounding concurrency’ in cell populations: the snow-balling effect of ever increasing numbers of cells engaging in some activity, and populations of which exhibit positive and negative feedbacks on one another. Expansion regions are used to indicate multiple invocations of some activities. The region marks an area of the diagram where actions occur once for each item in a collection comprising the region's input. Inputs and outputs are denoted using small adjoining boxes. Figure 6*a* shows an example expansion region: the output of A leads to multiple instances of B leading to C, all of which must complete before activity D commences. A shorthand compact notation for a single activity being invoked multiple times is shown in figure 6*b*.

Figure 6*c* depicts three different applications of expansion region concepts to a simple hypothetical perspective, where an immunization leads to the maturation of many DCs, each of which go on to activate (‘prime’) many T cells. The first example, (i), shows the encapsulation of expansion regions, reflecting the fact that each of many DCs primes many T cells. However, when applied to a larger and more realistically scoped perspective, entailing many more compounding concurrent activities, this notation potentially adds significant complexity to the diagram. Furthermore, it is not clear where the termination of the expansion region should lie for perspectives containing cyclic paths. The second example, (ii), makes use of the compact notation. The output collections have been omitted in an attempt to indicate that activities following an expansion region do not wait for all the invocations of the region to complete. However, while UML syntax allows for a region's inputs and outputs to differ in number, its semantics dictate that the region then acts as a filter, with some tokens of execution being dropped [10]. This is not the intended interpretation. The third example, (iii), makes use of multiple outputs from a region containing only a single input, to indicate that many invocations may follow the activity. Although (iii) most closely matches the intended concept, it fails to do so satisfactorily. The undesired implication of a sequential transfer of control remains: that *no* T cells can commence priming until *all* DCs have matured as a result of immunization. These activities are in fact ongoing and overlapping.

Expansion regions offer no satisfactory means to represent the compounding concurrency of biological systems, and this motivates the creation of our propagating relationship, discussed below in §5.

### 4.2. Class diagrams

**Figure 7.**
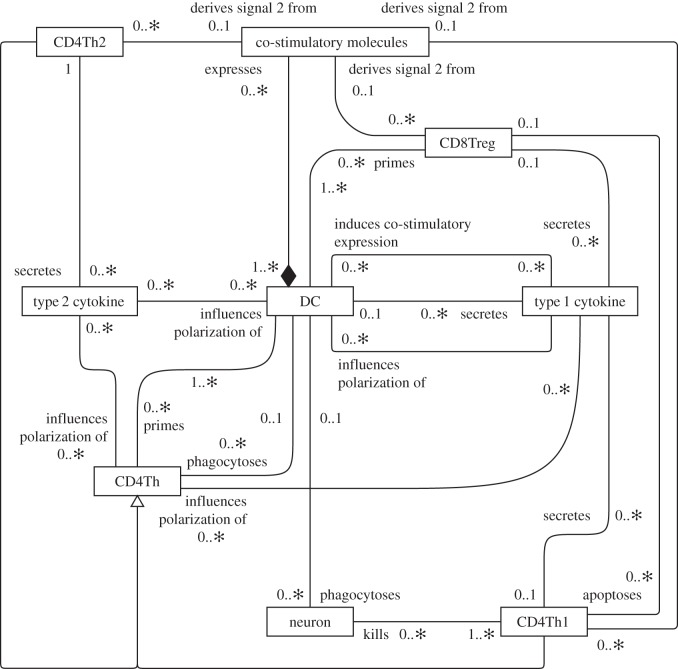
UML class diagram depicting relationships between entities of the domain model responsible for switching the CD4Th1 response to a CD4Th2 response. Adapted from [4].

To complement the role of activity diagrams in capturing the perspectives of our domain model, we investigate the ability of class diagrams in specifying the quantities of entities that engage in particular activities. For example, figure 7 denotes a class diagram of the cells and molecules involved in switching the CD4Th1 response to a CD4Th2 response. The diagram highlights relationships between entities such as cells, receptors and soluble signalling molecules (cytokines), and the number of these entities that may engage in these relationships. The central component of this response is the DC, which activates T cell populations.

#### 4.2.1. Relationships between entities

In our example (figure 7), the generalization relationship has been used in only one context in perspective class diagrams, denoting that both CD4Th1 and CD4Th2 cells are examples of CD4Th cells. With respect to our domain modelling requirements, generalization provides little meaning at the biological level as cells and molecules have no notion of generalization. However, these concepts can prove useful when attempting to abstract behaviours into a domain model, where they can serve to reduce the number of associations depicted. Generalization is a fundamental aspect of quality coding practice, but this is outside the scope of this paper.

**Figure 8.**
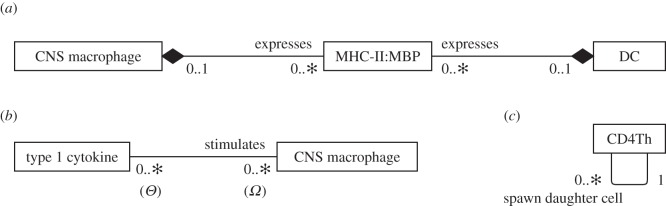
Select relationships between entities in the EAE domain model. (*a*) The use of UML class diagram composition relationships depicting mutually exclusive expression of MHC : peptide complexes on cells. (*b*) The relationship between type 1 cytokines and CNS macrophages. The cardinalities have been labelled ‘*Θ*’ and ‘*Ω*’ to facilitate discussion in the text. (*c*) The relationship that a CD4Th cell has in producing additional instances of itself.

UML composition relationships capture well the mutually exclusive manner in which cell-surface molecules (such as MHC-II : MBP) can be expressed by only one cell instance, even when many cell types are capable. This is demonstrated in figure 8*a*. Composition relationships have ‘no sharing’ semantics that entail only one composition relationship may be exercised by a particular entity instance at a time [10]. Furthermore, the destruction of the ‘owner’ instance entails the destruction of all ‘owned’ instances; the ingestion and processing of a cell by a DC entails the destruction of all cell-surface receptors it expresses. However, this relationship is not appropriate for modelling cell-signalling molecule relationships. A molecule instance can only be secreted by a single cell instance, yet the destruction of the cell has no bearing on the molecule's existence. Secretion by only a single cell could be expressed as a constraint using notes; however this information has instead been stated in text accompanying the diagrams (see the supporting information of [4]) as the number of notes would clutter the diagrams.

The complex, dynamic and stochastic nature of biological cells entails that most relationships depicted on class diagrams have ‘0..∗’ cardinalities. Cells may bind and disassociate with many other cells during their lifespans; some might do this many times, others never. The remainder tends to have ‘0..1’ cardinalities, reflecting secretion and expression constraints of the biology. The classes are also highly connected, reflecting the complex nature of systems such as EAE that involve highly connected biological entities. This does not aid their readability.

#### 4.2.2. Ambiguity in class diagrams

It is questionable that the information provided by these class diagrams is useful when trying to capture dynamic nature of the domain model perspectives. Class diagrams are ideal for depicting static relationships, but the inherently dynamic nature of biological systems can lead to ambiguity when interpreting them, for example the time frame over which an association with a cardinality holds is not necessarily apparent. Cardinalities indicate the number of instances that engage in relationships over time, and the ambiguity arises where there are multiple relevant temporal aspects to a relationship.

To illustrate the time ambiguity of cardinalities, figure 8*b* shows the relationship between a molecule called ‘type 1 cytokine’ and a CNS macrophage cell. The molecule ‘stimulates’ a CNS macrophage, and both ends of the relationship are annotated with ‘0..∗’ cardinalities. The relationship between a type 1 molecule and a CNS macrophage, and how different aspects of this relationship result in the requirement for different cardinalities, can be summarized as follows:

— Over the course of its lifespan, a CNS macrophage instance may perceive any number of type 1 cytokines; some instances will perceive none, others a great many. This suggests that cardinality ‘*Θ*’ in figure 8*b* should be ‘0..*x*’, 0 ≤ *x* ≤ ∗.
— CNS macrophage stimulation requires exposure to a threshold concentration of type 1 cytokine, suggesting cardinality ‘*Θ*’ should be ‘*x*’, 0 < *x* < ∗. However, this fails to adequately represent CNS macrophages that are not stimulated during their lifetimes, which is better reflected by ‘0..*x*’.
— In this model, type 1 cytokine instances are not destroyed through cellular interaction and they may disengage and interact with other cells. This suggests a ‘0..∗’ cardinality for ‘*Ω*’ (figure 8*b*). However, a cytokine instance can be perceived only by a single cell at any single point in time, suggesting a ‘0..1’ cardinality.

A further example of ambiguity concerning the time frame over which relationship cardinalities apply may be found in figure 8*c*, which denotes cellular proliferation. It is valid to interpret cardinalities as the number of instances that engage in the relationship at any single point in time. Under this interpretation, the relationship indicates that a cell provides any number of daughter cells simultaneously, for example that it may divide five ways to produce five daughter cells. However, this is biologically inaccurate: only single daughter cells are produced at a time through cell division. The relationship is intended to describe the fact that a CD4Th cell has only a single parent cell, but that a parent cell may spawn any number of daughter cells during its proliferative cycle.

Like many complex biological systems, immunology is a highly dynamic and stochastic domain, cells and molecules engage and disengage frequently. Some instances engage in particular relationships frequently, whereas others never engage. There are many temporal aspects of the relationship that are relevant to the domain, and UML class diagrams present no means to reflect them all; each end of a UML relationship is permitted only one cardinality. There are no UML guidelines on the temporal scope to which a cardinality applies, and further, in immunology this is specific to the particular relationship. Modelling dynamic and stochastic systems using a notation intended for specifying static relationships can lead to ambiguities that obfuscate rather than clarify. Coupled with cardinalities that are predominantly ‘0..1’ or ‘0..∗’, and the highly connected nature of classes, we do not consider the class diagram a particularly informative or comprehensible formalism for capturing perspectives in a domain model.

### 4.3. Sequence diagrams

**Figure 9.**
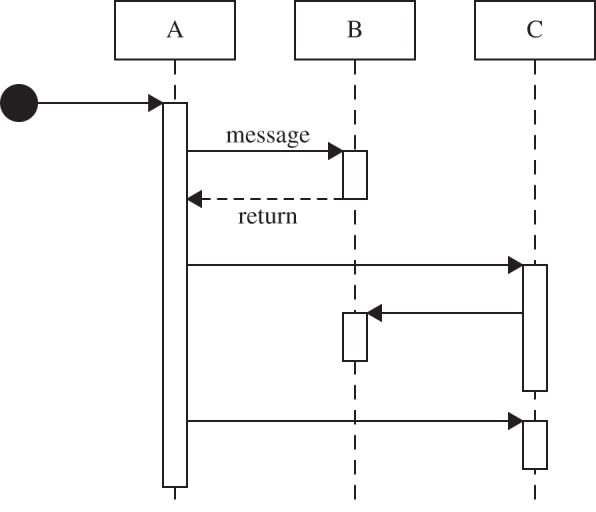
An example UML sequence diagram, depicting the messages passed between participants A, B and C.

Given the timing intricacies involved in capturing the domain model perspectives that we have explored with activity and class diagrams, UML's sequence diagrams offer an alternative notation for expressing how dynamic system entities collaborate. A simple example sequence diagram is presented in figure 9. Like activity diagrams, sequence diagrams allow for the depiction of concurrent and alternative paths of execution. However, their syntax contains an inherent single thread of control such that time is represented linearly along only one dimension, preventing the expression of cyclic groups of events that appear throughout the biology. Furthermore, the vertical notation depicting ‘lifelines’ during which messages are passed between participants implies that participants pass control to one another and wait until other lifelines have completed before they are themselves able to continue. This is in contradiction with biological reality, where entities are entirely concurrent and do not typically block one another from being able to interact with one another. This may be overcome using multiple, coupled sequence diagrams, each of which captures a specific subset of the overall dynamic being modelled; the behaviour specified in each sequence diagram is invoked when its preconditions are met, as exemplified in [31]. In this manner, a perspective could be represented by a collection of sequence diagrams. However, as only a single activity diagram can fulfil the requirements for modelling a perspective, as laid out in §2, we consider activity diagrams the more appropriate format for the current EAE case study.

### 4.4. State diagrams

To fulfil the lowest level of our domain modelling framework outlined above in our requirements, we have used state diagrams to capture the single-entity dynamics of domain entities in a natural and useful way. In particular, transition guards between states provide a way of modelling logical, probabilistic and time-dependent concepts. In the state diagrams presented here, the following notation has been used in transition guards:

— ‘&’ and ‘|’ indicate logical conjunction and disjunction respectively.
— ‘*δ* (condition)’ indicates probabilistic events.
— ‘*λ* (condition)’ indicates events that occur after some period of time.

While these are well-established concepts in modelling, the UML 2.4.1 does not specify a syntax for their expression in state machine diagrams [32,33]; hence, we define our own.

**Figure 10.**
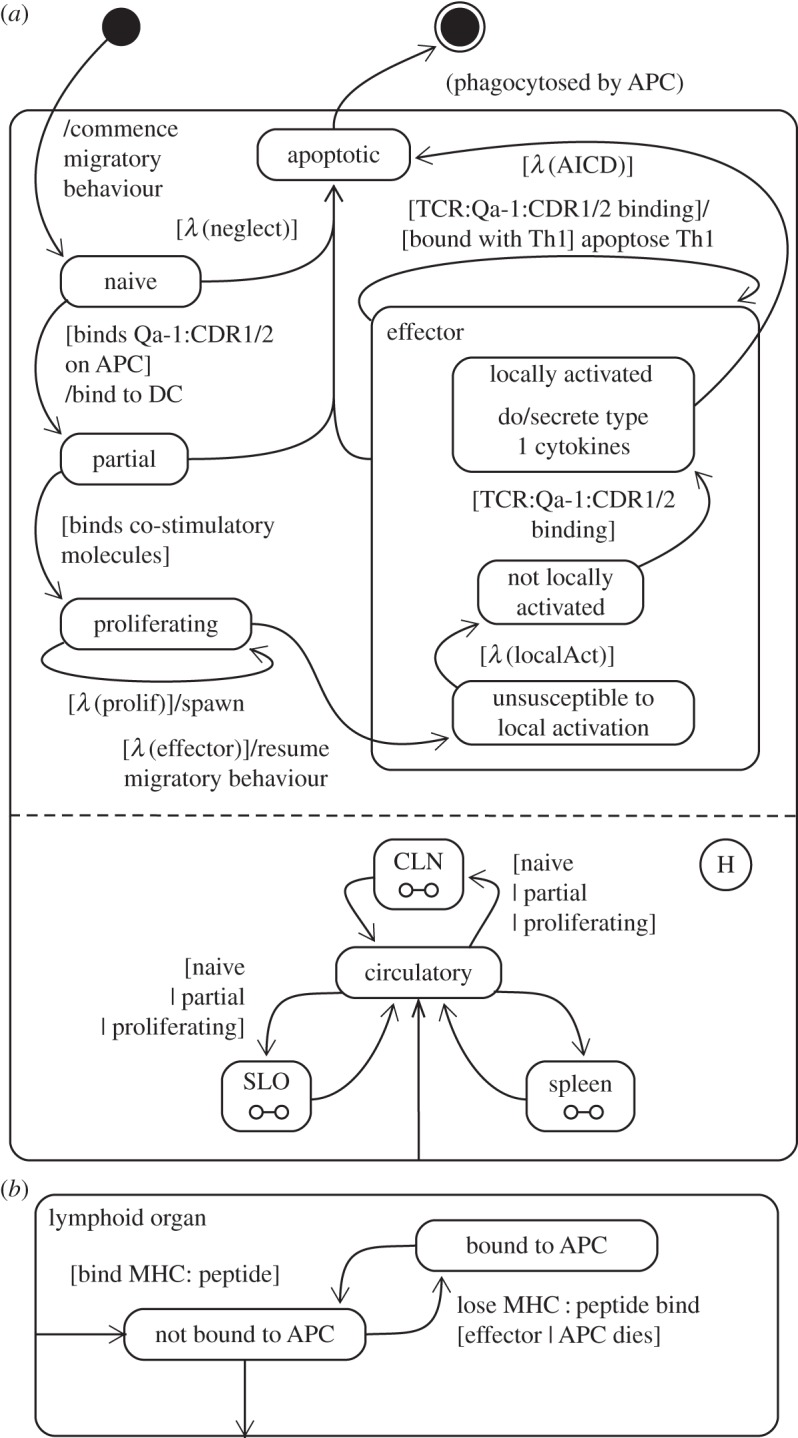
(*a*) State machine diagram depicting the dynamics of CD8Treg cells. (*b*) Decomposition of the lymphoid organ states: the SLO, CLN and spleen. Adapted from [4].

**Figure 11.**
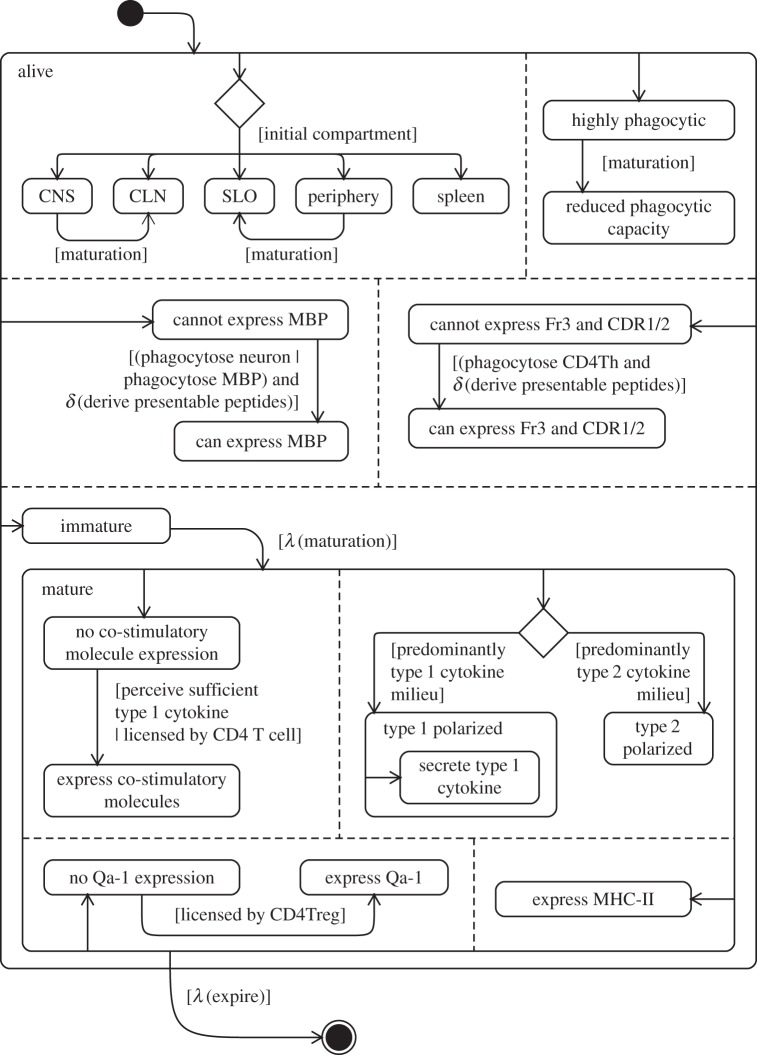
State machine diagram depicting the dynamics of dendritic cells. Adapted from [4].

Most biological cells, such as T cells or DCs, have complex multi-dimensional dynamics (e.g. receptor expression levels). Furthermore, these dimensions are not always completely orthogonal. These features of cellular dynamics can complicate their depiction as state machine diagrams: expressing high-dimensional partially orthogonal information on a two-dimensional diagram is challenging. For example, figure 10 captures the dynamics of CD8Treg cells as a state machine diagram. The locations in which CD8Treg cells may reside are depicted as a mutually exclusive set of states that are orthogonal to the rest of the cell's dynamics, such as its states of maturation. However, these sets of states are not entirely mutually exclusive: state transitions resulting from receptor binding (e.g. TCR:Qa-1:CDR1/2 binding) can occur only in spatial compartments where the corresponding receptor targets are found. In this case, the targets (Qa-1:CDR1/2 expressed by DCs) are not found in the circulatory system. This can be determined through cross-reference with other state machine diagrams (for DCs, figure 11; and CNS macrophages, see supporting information of [4]). However, depicting these constraints directly on the CD8Treg cell state machine diagram would increase its complexity and hinder its comprehension, which undermines the goal in presenting a transparent and informative domain model. UML state machine diagram transition guards have been employed in dealing with partial orthogonality. For example, state transitions pertaining to the cell's migration into spatial compartments are guarded based on the cell's state of maturation; effector CD8Treg cells cannot enter the cervical lymph node (CLN), spleen or secondary lymphoid organ (SLO) compartments.

State machine diagrams are effective in capturing orthogonal and categorical concepts where states do not overlap. Molecular expression in the present domain model has been represented in this manner: a DC (figure 11) either expresses MHC-II:MBP in sufficient quantities to activate (prime) T cells, or it does not. However, this is an abstraction; real DCs express constantly varying levels of MHC, low while immature and increased following maturation [34]. T cells in certain developmental stages require more MHC-receptor bindings to be stimulated than others. Fine-grained quantities such as levels of molecule expression or rates of molecular secretion cannot be captured using UML state machine diagram notation, and require mathematical or textual accompaniment.

UML provides no explicit syntax for representing probabilistic or temporal parameters, such as transitions that may occur only after some period of time has elapsed, or transitions that occur probabilistically or at some rate. These are highly relevant to stages of a cell's life cycle, and nearly every cell's state machine diagram in the EAE domain model contains such a notion. We have addressed this by employing guard statements of the form [*λ* (condition)] and [*δ* (condition)] to concisely indicate the passage of sufficient time or transitions that occur probabilistically. Bersini *et al.* [35] have represented probabilistic events as guards in combination with a state machine diagram choice node. In our present model, choice is to either remain in the current state, or traverse to another, and as such we have omitted the choice node in our diagrams.

We have found it useful to create state machine diagrams containing single states with no transitions, and which are orthogonal to other states. An example of this may be found in figure 11, where a mature DC is always capable of expressing MHC-II. It is unconventional for states on state machine diagrams to exist in isolation such as this; however, it is relevant and informative in communicating immunological concepts such as receptor expression.

**Figure 12.**
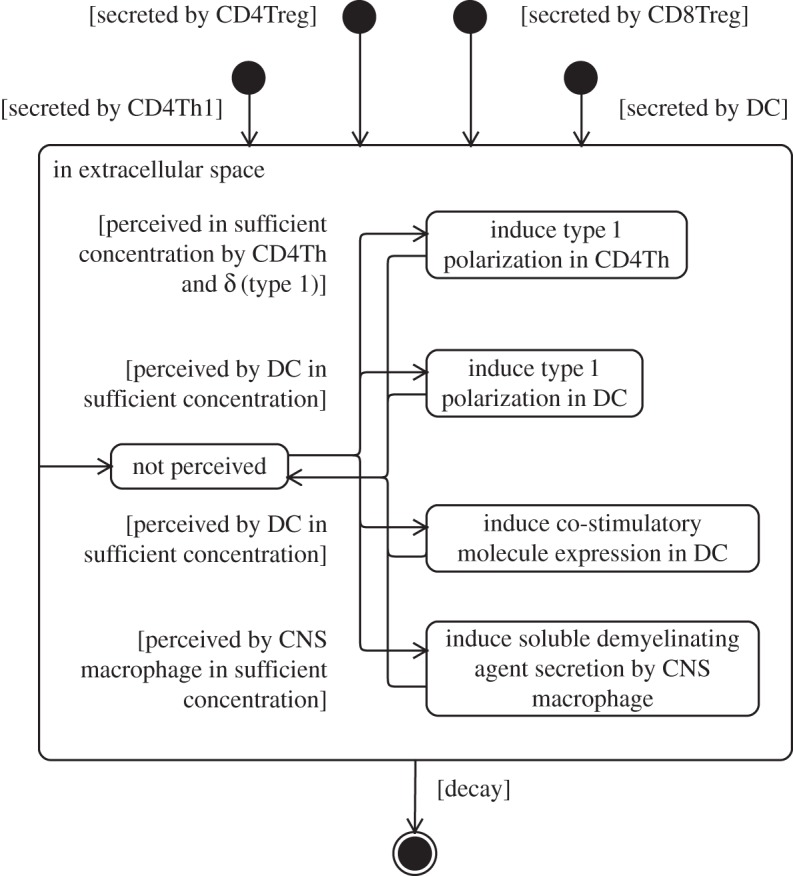
State machine diagram depicting the dynamics of a soluble signalling molecule (type 1 cytokine), and its influence on other cells of the domain model. Adapted from [4].

It has also proven useful to create state machine diagrams containing states that do not actually describe an entity's internal state. It may be argued that the spatial compartment in which a cell resides is not part of its internal state, yet it has proven informative to indicate on cellular state machine diagrams which compartments the cell may reside in, and the conditions necessary for it to migrate elsewhere. A further example is found in figure 12, which depicts the influence of a signalling molecule on the cells that can perceive it. The diagram indicates the conditions necessary for their perception to induce behavioural changes in the various cells of the system. The influence a molecule has on another entity of the domain is not part of its internal state, yet it is informative to consolidate this information onto a single diagram.

## 5. Modifying activity diagrams

**Figure 13.**
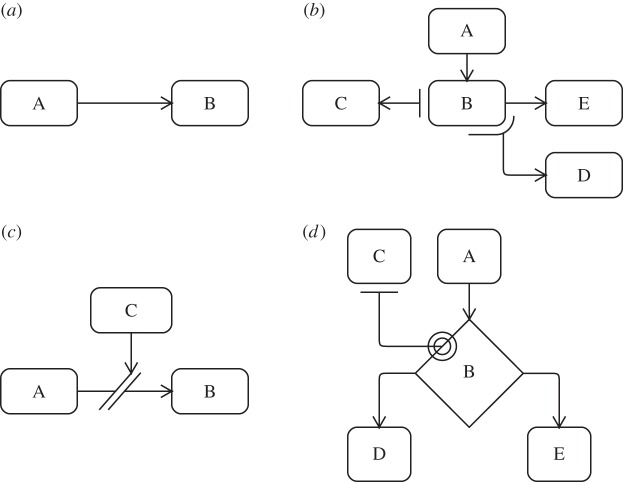
The types of extended relationship used in activity diagrams. (*a*) Sequential relationship, an arrow. (*b*) Propagating relationships, and arrow not joined to the originating box. (*c*) Interruptible relationship, diagonal lines intersecting an arrow. (*d*) Contributory relationship, circles.

Standard activity diagram notation was shown above to be a useful tool for capturing the system-level perspectives of a complex biological domain. However, there are a number of important biological concepts and modes of behaviour that are not easily expressed using this standard notation. Figure 13*a* depicts the standard sequential state transition of a UML activity diagram. In biology, an entity (e.g. a cell) undertaking some action does not necessarily become inactive afterwards, as implied by the arrow; it may continue to interact with others in a manner not depicted on the diagram. Here, we present three modifications that fill this gap: the *propagation* relationship, the *interruptible* relationship and the *contributory* relationship.

*Propagation relationship*: figure 13*b* depicts a propagation relationship, which depicts an entity performing some activity which has a number of consequences elsewhere, without necessitating that the former activity terminates. In the example, activity A leads to activity B. Activity B results in activities C and D taking place, whereas B continues. Hence, activities C and D may be considered new tokens of execution, and they may occur any number of times as a result of B taking place. At some point, B terminates, leading to activity E, at which point no more occurrences of C and D are generated. This relationship might be used to represent cellular division, with a mother cell generating a number of daughter cells, before engaging in some other activity.
*Interruptible relationship*: figure 13*c* depicts a relationship that can be downregulated or interrupted. Ordinarily, activity A leads to B. However, the occurrence of activity C can partially, or fully, prevent transitions from A to B. For example, C might represent secretion of a soluble factor that interferes with a population represented in A having an effect on another population represented in B. The interference is not necessarily absolute, it may be partial.
*Contributory relationship*: figure 13*d* depicts a contributory relationship. Activity A leads to decision B, after which either activity D or E will take place. The decision is influenced by activity C, which did not itself instigate it, and represents a separate token of execution. In this example, the influence of C on B is propagative, and as such, a single activity C can influence many decisions B.

**Figure 14.**
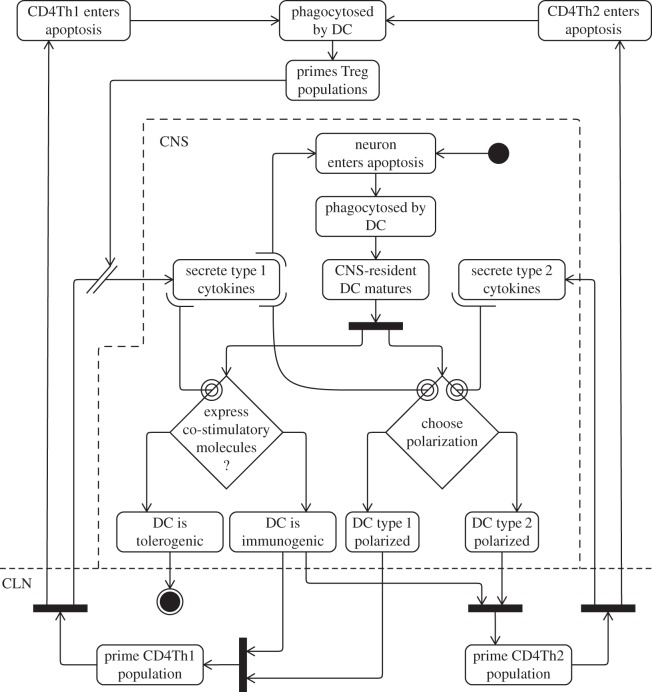
UML activity diagram depicting the cellular interactions and events that lead to a type 2 deviation of the immune response. Adapted from [4].

Examples of these three relationships are shown in figure 14, which depicts biological perspective expressing the switch from an autoimmune-inducing CD4Th1 immune response to a less harmful CD4Th2 response. CD4Th1 and CDTh2 responses cross-regulate and suppress one another through the actions of both soluble signalling molecules (cytokines) and by influencing the actions of DCs. DCs ingest dead neurons, and the particular balance of signalling molecules in their local environment influences their subsequent receptor expressions and polarizations. This is depicted by a combination of our propagating and contributory relationships: cells secrete many molecules, and these have multiple influence on other cell populations. A DC's polarization and levels of receptor expression influence whether they activate CD4Th1 or CD4Th2 cells, or neither (for tolerogenic DCs). It is in this way that both these T cell populations suppress one another: by secreting the signalling molecules influencing DC behaviours. Recovery from autoimmunity is mediated partially by CD8Treg cells which kill CD4Th1 cells, depicted as an interrupting relationship, and hence prevent the secretion of signalling molecules favouring CD4Th1 activation.

## 6. Discussion

We have presented a case study in constructing a model of a complex biological domain using aspects of the UML. The modelling was driven by the requirement to capture biological behaviours as they are understood by the relevant research community. This requirement led us to outline a multi-level modelling framework where levels are populated with multiple diagrams. Each level captures a behavioural subset: a system-level overview that relates the key model entities and behaviours to the observed biology; system-level perspectives that describe how cellular interactions lead to particular biological behaviours and consequences; and single-entity-level diagrams describing the dynamics of entities (such as cells) and their interactions in the environment. The collection of diagrams (and accompanying text) from each of these three levels forms our overall domain model of the biology. We believe that this multi-level framework is generalizable to modelling other complex biological systems.

Based on our modelling framework, we explored the utility of the UML's diagrams. We have not shown examples of using all the UML's diagrams, but instead focused on those that have been, or appeared to be, most useful. We found activity diagrams to be a natural means of capturing the mid-level perspectives, and state diagrams are a similarly natural way of capturing the single-entity dynamics. Class diagrams and sequence diagrams have not proved to be particularly useful for the highly dynamic (in terms of numbers of entities and timings) nature of EAE, the biological system used as the case study. For biological systems with more static structures, class diagrams may prove more useful; however, most biological systems are inherently dynamic, which leads to the nonlinear emergent behaviours that characterize them. At the top, system overview level of modelling, we could not find a UML diagram notation able to capture the required concepts. Instead, we resorted to using specialized informal diagrammatic notations, such as the research context diagram, to capture the required biological concepts.

While activity diagrams were shown to be useful for capturing biological perspectives, their inability to unambiguously represent a number of core biological concepts led us to propose several modifications to the activity diagram syntax. These modifications, the propagating, interruptible and contributory relationships, help us capture the action of entity populations in the context of activities. Their expressiveness enabled us to better represent the biology as it is understood by the research community, and thus model the action of large populations of entities, which often result in the emergent behaviours seen across biological systems.

Our application of activity and state diagrams often differs from ‘conventional’ use. Along with our modified activity diagram syntax, biological feedbacks and compounding concurrency have manifested as activity diagrams with no terminating state, which, though unconventional, do express the intended concepts. The creation of state machine diagrams expressing the effects that a molecule can have on cells is also unconventional, as these effects do not represent state; however they are useful in conveying the biology. The primary focus of our domain modelling is not to obey the conventions of UML, but to clearly communicate the biology. This may entail using UML in a non-standard way, and the characteristics of biological systems often make this desirable. As long as the modifications to usage are explained and understood by the parties using the diagrams, we believe this is an acceptable way of using the UML notations. Similarly, where no natural fit to UML exists, specialized diagrams with less stringent semantics can be used, so long as they are well explained.

Constructing the EAE domain model has been an incremental task following the CoSMoS process, our wider strategy for engineering high-quality simulations for use in exploring complex system domains, explored in further detail in the electronic supplementary material and in [6–8]. The domain model focuses purely on biological concerns, it captures hypotheses, abstractions and assumptions, and communicates the biology that a simulation represents in a clear and coherent manner. It intentionally does not consider implementation-specific details. Rather, a subsequent CoSMoS modelling phase translates the domain model into a software specification (termed the *platform model*), explicitly recording implementation-specific concerns, assumptions and constructs. An example of how domain and platform models differ in purpose and scope may be found in the electronic supplementary material. From the platform model, an executable simulation platform is constructed, which can be used to run experiments and evaluate hypotheses. The CoSMoS process is iterative, and where simulation results fail to adequately capture the biology, each of the modelling phases is revisited. This commences through consultation with a domain expert where hypotheses, assumptions and abstractions recorded in the domain model are revisited. Literature survey is conducted to further identify significant aspects of the biology, and the domain model diagrams are amended as inconsistencies arise and additional biological information emerges. These amendments are then filtered through the rest of the CoSMoS process, and the revised simulation is re-evaluated. After a number of iterations, the domain model (and the simulation) is able to capture the relevant biological concepts to the satisfaction of the domain expert. It is interesting to note that the process of developing the domain model can facilitate reasoning about the biological domain as the modelling process forces one to produce a consistent, logical description of the behaviours, which in turn can throw up contradictions or gaps in biological knowledge. This can be a rewarding experience for all parties involved in the modelling. The present EAE domain model became a shared resource for discussions between the domain expert and simulation engineers for developing an implementation of the model (a simulator), running simulation experiments and interpreting simulation results with respect to the biology. The simulator that resulted from the EAE domain model is called artificial murine multiple sclerosis [4,18,19], and as a result the domain model records the biology that underpins the simulator and its results.

There exist technologies for creating executable diagrammatic models, for example, using IBM's rational rhapsody to create executable state-machines [27,30,36], using live sequence charts in conjunction with the play engine to create executable sequence diagrams [29,31], or generating ordinary differential equations from UML state machine diagrams [35]. These technologies are well suited to platform model and simulation implementations, and the manner in which domain models can be translated into executable software specification using the aforementioned technologies warrants further investigation.

The domain modelling discussed here is a vital element of the CoSMoS process. Other elements of CoSMoS include providing a structured argument laying out evidence to support a simulation's fitness for purpose [37]; statistically analysing a simulation to identify criticalities and sensitivities in components and pathways, and considering these in the context of the biology [38,39]; providing automated approaches to calibration [40]; and maintaining close ties with expert biologists in the fields being modelled [5].

Appreciating a simulation's biological model is essential for correctly interpreting its results, and understanding their contribution in the wider biological research context. Effectively communicating the biological model that a simulation captures is vital for a healthy simulation-based biological research field. The UML helps us to capture and communicate this model. Core to the process of science is the requirement that results be reproducible by third parties. This has culminated in calls to make research simulation code public, and arguably open source [41]. A clear and concise model of the biology that a simulator captures should help open source science to succeed and facilitate the validation of the simulators.

Computational modelling and simulation techniques have far-reaching potential in biological fields such as immunology. For instance, they can be used to gain insights into disease manifestation, consolidate data, and thereby help identify and evaluate promising pharmaceutical targets [42]. Simulations of human beings can be parametrized to particular patients, reflecting their medical history and current situation, and be used to explore and optimize potential disease treatment strategies [43]. However, it is critical that simulation results are demonstrated to be appropriate representations of the biology. Simulators, as man-made artefacts, have as much potential to mislead as they do to enlighten. Rigorous domain modelling is one important facet to a more principled biological simulation field.

## Supplementary Material

Supporting Materials
