# Supplementary material for: Modelling biological behaviours with the unified modelling language: an immunological case study and critique
Source: J R Soc Interface. 2014 Oct 6;11(99):20140704. doi: 10.1098/rsif.2014.0704 (PMC4233755; doi:10.1098/rsif.2014.0704)
Supplement: Supporting Materials [file rsif20140704supp1.pdf]

Supporting Materials

Dr. Mark Read

July 25, 2014

1 Overview of the CoSMoS process

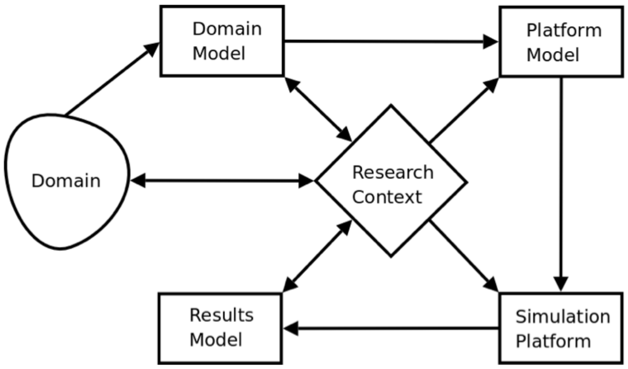

Figure 1: An overview of the artefacts comprising the CoSMoS process, and the flow of information between them. Reproduced from [1].

The CoSMoS process (Complex Systems Modelling and Simulation infrastructure)[1] is a generic process for complex system simulation, providing guidelines and techniques for the scientific investigation of complex systems domains through use of simulation. Full details of the process may be found in [1], and it is further explored in the context of particular biological domains in [2, 3]. We provide here only a brief overview.

The CoSMoS process views simulation-based complex systems research as a necessarily interdisciplinary endeavour between scientific experts in a complex system domain, and engineers skilled in the construction and use of simulations. The process has been developed to add rigour to simulation-based research, making explicit artefacts typically dealt with implicitly, and thereby promoting higher quality and more transparent research. Figure 1 provides an overview of the process and its artefacts.

The *domain* represents the complex system of interest, in the present case it comprises the EAE disease model studied by Vipin Kumar's lab. The *domain model* is a non-executable model of the domain. It may encapsulate processes, structures, emergent properties, theories & hypotheses, and possibly data concerning the domain of interest. It is purely focussed on scientific aspects of the domain, and is explicitly free of any implementation concerns. As outlined in the accompanying manuscript, it is intended to promote a thorough exploration of the domain, highlight inconsistencies and information gaps in the scientific understanding of the domain, make explicit the assumptions necessitated thereby, and provide a comprehensive and consistent view of the domain.

The *platform model* constitutes the software specification for the simulation platform. A domain model can be simulated in a number of different ways, and each possible strategy for implementation is accompanied by its own set of assumptions. Furthermore, the hypotheses and emergent properties explicitly captured in the domain model should not appear explicitly in any software specification; these emergent properties should be observed as high level behaviours emanating from the simulation in the same manner that they do in the domain, and hypotheses are to be tested based on simulation results. Neither should be directly coded into the simulation. All these reasons necessitate a separation of domain and platform models; the former dealing with scientific concerns in the domain, the latter with issues relating to implementation.

[1] *http://www.cs.york.ac.uk/nature/cosmos/*

The *simulation platform* is the software that simulates the domain. The CoSMoS process does not explicitly dictate how the simulation platform is constructed from the platform model, but it recommends that established software engineering principles, for example testing and agile software development, are adhered to. The process does not dictate exactly which software engineering principles be observed, it states only that simulation engineers should take steps to ensure high-quality, well-documented and bug-free code be developed using appropriate measures given the problem at hand.

The *results model* encapsulates observations and understanding that stems from the simulation: simulation dynamics, recorded data and statistics, and observations. The manner in which the domain model reflects the domain is mirrored in the results model's reflection of the simulation platform.

The *research context* captures the overall context in which the simulation-based research is being conducted. This includes the motivation for the research, the questions that are to be addressed, and requirements for evaluation of success and validation of results.

The CoSMoS process is intended to be an iterative process, with all of the artefacts undergoing potential modification between iterations. An iteration is envisaged to comprise three stages, *discovery* where the domain model is modified to reflect further investigation of the domain or changes to the context in which research is to be conducted; *development* in which the platform model and simulation platforms are updated to reflect the modified domain model; and *exploration*, where simulation experiments are performed.

Though it appears prescriptive in nature, the process is not intended to *force* researchers to explicitly undertake all iteration phases and maintain all artefacts. Rather, the process highlights that these phases and artefacts are inherent in any simulation endeavour, and prompts their consideration. How much effort is vested in explicitly undertaking or maintaining aspects of the process is a judgement call to be made by the investigators, and is informed by the complexity of the problem at hand, its criticality, and the rigour felt appropriate given the scientific questions being addressed.

With respect to the domain model, the CoSMoS process does not specify any particular format, formalism, or even the exact details that should constitute the model. It only defines the concept, and how it links to other artefacts. The present manuscript provides more specifics on domain modelling for a particular class of biological problems. The framework and use of UML that we present should not be interpreted as prescriptive for those following the CoSMoS process, it provides only an example method for domain modelling. There will be other methods, and investigators should approach domain modelling in a manner most appropriate to the problem at hand.

2 Domain Model Assumptions

The domain model (DM) is intended to capture the assumptions made of a biological system in creating a consistent abstract model of it. It is impossible to record everything that is abstracted, as the field of Biology is constantly expanding, and this exercise can theoretically distil down to the level of organic chemistry, physics, and thereafter quantum mechanics. Instead, a DM should be regarded as a record of assumptions in the sense of what *is* represented, rather than what is not.

The biological entities represented in a DM in actuality represent a great many biological factors at an abstract level. A domain model, or simulation derived from it, cannot be used to examine the dynamics or contributions to a biological phenomenon of biological components that have been abstracted into the same logical entity.

Although an exhaustive list of abstractions is impossible for the reasons outlined above, we provide the following list of key abstractions for illustration.

- The onset, persistence and recovery of EAE can be adequately described based on the activities occurring within a single peripheral lymph node, the circulatory system, spleen, central nervous system and a single cervical lymph node.
  Consequence: DM, and any simulation resulting from it, cannot be used to investigate the role of any other spatial compartments in the dynamics of EAE.

- Intracellular signalling cascades have not been explicitly represented. Instead, the behavioural changes that result from extra-cellular signalling events (mediated through, for example, cytokines and receptors) have been represented.
  Consequence: The dynamics and contributions of aspects of intracellular signalling cascades to cellular behaviours can not be examined using this DM, or any simulation derived from it.

- The activities of CD4Th17 cells is sufficiently similar to those of CD4Th1 cells that they have not been explicitly represented. The DM's CD4Th1 cells abstract the behaviour of both CD4Th1 and CD4Th17 cells in the real biology.
  Consequence: DM, and any simulation resulting from it, cannot be used to examine the individual contributions of CD4Th1 and CD4Th17 cells to establishment and persistence of EAE.

- The actions of IL-2, INF-γ, IL-12, IL-17 are sufficiently similar to be abstracted into a single cytokine type, *type 1 cytokine*.
  Consequence: DM, and any simulation resulting from it, cannot be used to examine the individual roles of these cytokines in EAE.

- The actions of IL-4 and IL-10 are sufficiently similar to be abstracted into a single cytokine type, *type 2 cytokine*.
  Consequence: DM, and any simulation resulting from it, cannot be used to examine the individual roles of these cytokines in EAE.

- Demyelination is abstracted as neuronal apoptosis. Phagocytosis of 'apoptotic' neurons leads to MHC:MBP presentation on antigen presenting cells.
  Consequence: DM, and any simulation resulting from it, cannot examine the specific contributions of the demyelination process and the specific behaviour of demyelinated neurons (as opposed to fully functioning, or apoptotic) on the dynamics of EAE.

- Microglia and macrophages residing in the CNS are sufficiently similar in function to be abstractly represented as a single cell, the *CNS macrophage*.
  Consequence: DM, and any simulation resulting from it, cannot be used to examine the individual roles that these cells have in EAE.

3 Domain Model as Different from the Platform Model

We consider here how a platform model is different from a domain model.

The purpose a domain model is not to provide a comprehensive implementation specification for a simulation. Rather, it is intended to highlight hypotheses, abstractions and assumptions, and for communicating the biology that a simulation represents in a clear and coherent manner.

A simulation specification will make additional assumptions from those present in a domain model where insufficient biological detail is available, and provides specifics of how implementation is to be accomplished. This can be illustrated by comparing elements of our EAE domain model with its implementation described in [4]. Specifically, figure 11 of our manuscript depicts the dynamics of dendritic cells (DC) in our domain model, highlighting how DCs migrate from the periphery into the SLO upon maturation, an event which leads to the establishment of autoimmunity. Neither it, nor any other element of the domain model, indicates how many DCs undergo this migration nor how quickly; this information is not known, but must be provided in the software specification. Figure 2 (below), reproduced from [4], illustrates how the establishment of autoimmunity is to be implemented in ARTIMMUS: through the periodic creation of MBP-presenting immunogenic type 1-polarised DCs in the SLO compartment. It explicitly references simulation parameters, and describes how they together specify a linearly decreasing number of DCs to be created over time. The simulation specification makes further assumptions of the domain model such as omitting the periphery from explicit representation. There are many alternative ways in which immunization for EAE could be implemented based on information contained in the same domain model, each necessitating different assumptions. Most importantly, the domain model contains information that should not be present in the simulation specification. For instance, it describes how a cascade of cellular interactions, starting with immunization for EAE resulting in MBP-presenting DCs migrating from the periphery to the SLO compartment, culminate in damage to the central nervous system. High level overviews such as this are hypotheses, and must not be directly coded into the simulation; they represent the abstract outcomes we seek to observe from a simulation which explicitly codes only single-cell-level behavioural dynamics, thereby allowing us to evaluate our hypotheses. Someone wishing to understand the biology underpinning ARTIMMUS would not benefit from having implementation-level details obscuring the portrayal of purely biological concepts, and someone wishing to implement ARTIMMUS would require more specifics than is contained purely in the domain model. The domain model and simulation specification have different purposes, and are therefore two explicitly separate entities. Domain modelling provides the starting point for simulation specification.

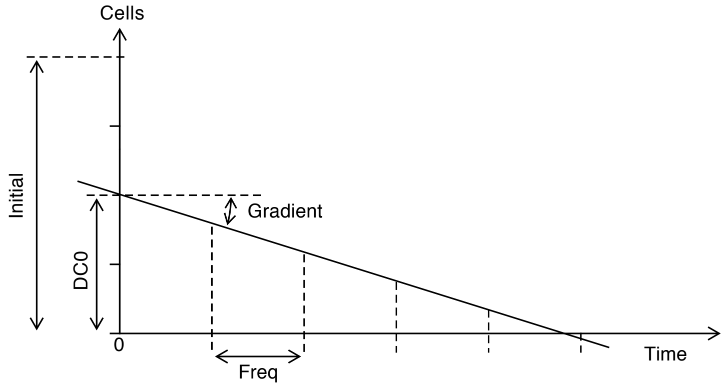

Figure 2: ARTIMMUS's immunization mechanism, and how it is parametrised. This comprises part of ARTIMMUS's simulation specification. The label "*Simulation_immunizationLinear*" has been omitted from parameter names. Immunisation for EAE is accomplished *in vivo* through the administration of MBP, PTx and CFA. These immunisation substances do not find explicit representation within the simulation, which instead represents immunisation through the appearance of MBP-presenting immunogenic type 1 polarising DCs in the SLO compartment. Hence, the periphery compartment of the domain model is not represented in the simulation specification, and is not implemented in ARTIMMUS. The immunisation mechanism is parametrised through 4 parameters: *Simulation_immunizationDC0*, *Simulation_immunizationLinearFreq*, *Simulation_immunizationLinearGradient*, and *Simulation_immunizationLinearInitial*. The last specifies the number of immunisation DCs placed into the SLO compartment at time zero, as a one-off event. The remainder parametrise a linearly reducing number of DCs that are added to the SLO periodically. The period is defined by *Simulation_immunizationLinearFreq*. *Simulation_immunizationDC0* and *Simulation_immunizationLinearGradient* describe the level of DCs inserted at time zero, and the rate of linear decay. Every *Simulation_immunizationLinearFreq* hours, the value described by these two parameters, given the current simulation time, is rounded to the nearest whole number of DCs which are then placed in the SLO. Reproduced from [4].

References

[1] Andrews PS, Polack FAC, Sampson AT, Stepney S, Timmis J. 2010 *The CoSMoS Process Version 0.1: A Process for the Modelling and Simulation of Complex Systems.* Technical report, YCS-2010-453: Department of Computer Science, The University of York.

[2] Bown J, Andrews PS, Deeni Y, Goltsov A, Idowu M, Polack FA, Sampson AT, Shovman M, Stepney S. 2012 Engineering simulations for cancer systems biology. *Curr Drug Targets* **13**, 1560-74. (DOI:10.2174/138945012803530071)

[3] Alden K, Timmis J, Andrews PS, Veiga-Fernandes H, Coles M. 2012. Pairing experimentation and computer modeling to understand the role of tissue inducer cells in the development of lymphoid organs. *Front Immun* **3**, 172. (DOI:10.3389/fimmu.2012.00172)

[4] Read M, Andrews PS, Timmis J, Williams RA, Greaves RB, Sheng H, Coles M, Kumar V. 2013 Determining disease intervention strategies using spatially resolved simulations. *PLOS ONE* **8**, e80506. (DOI:10.1371/journal.pone.0080506)
